# Extreme rainfall, flooding and malaria in the Sahara: outbreak analysis in Kidal, Mali 2024

**DOI:** 10.1186/s12936-026-05833-z

**Published:** 2026-03-06

**Authors:** A. M. Dolo, M. Cissoko, A. Teme, K. Keita, M. Sanogo, C. A. T. Traoré, M. Magassa, A. Koné, F. A. Roy, I. Sagara, J. Gaudart

**Affiliations:** 1 Malaria Research and Training Center (MRTC), FMOS & FAPH, USTTB, BP 1805 Bamako, Mali; 2 Aix Marseille Univ, IRD, INSERM, SESSTIM, UMR1252, ISSPAM, 13005 Marseille, France; 3 National Malaria Control Program (NMCP), BP 233 Bamako, Mali; 4 Directorate General of Health and Public Hygiene (DGHPH), BP 233 Bamako, Mali; 5 Kidal Regional Health Direction, Kidal, Mali; 6 Univ. Boni, Digital health & Innovation Research Unit, LaMIC-SD, Bobo Dioulasso, Burkina Faso; 7 Aix Marseille Univ, INSERM, IRD, SESSTIM, UMR1252, ISSPAM, APHM, BioSTIC, Biostatistic & ICT unit, 13005 Marseille, France

**Keywords:** Outbreak, Sahara, Malaria, Climate change, Extreme rainfall, Floods, Mali

## Abstract

**Background:**

Extreme rainfall caused severe flooding in Kidal, northern Mali, in 2024, raising concerns about the outbreak of malaria associated with climate change. The objective of this study was to describe this epidemic and the response implemented by national and regional authorities.

**Methods:**

A historical cohort study was conducted in the Kidal region, covering the entire population. Weekly malaria case data recorded by the national health system between May 2024 and February 2025 were analyzed in conjunction with precipitation data from the Tropical Rainfall Measuring Mission (TRMM, spatial resolution: 0.25°). The interventions included seasonal malaria chemoprevention (SMC), extended to children under 15 years of age, and deployment of mobile health teams in remote areas. The effect of these interventions on malaria cases per 1000 person-weeks was estimated using Generalized Additive models (GAM) applied to interrupted time series, taking rainfall into account. In addition, the spatial distribution of the population at risk was estimated using WorldPop population data.

**Results:**

Rainfall began in week 25 and continued for 15 weeks. The malaria epidemic began in week 30, peaked in week 39 with 1,014 cases, and lasted for 28 weeks, with 1.688 (95% CI 1.687–1.690) cases per 1000 person-weeks. Incidence was highest among children under five. The interventions implemented, using extended SMC and mobile health teams, led respectively to a significant reduction in morbidity (respective Standardized Incidence Ratios (SIRs) of 0.50; 95% CI 0.33–0.75 and SIRs = 0.48; 95% CI 0.28–0.82).

**Conclusion:**

Malaria outbreaks associated with flooding are becoming increasingly frequent in the context of extreme weather conditions. These situations pose a major challenge for malaria control programs and highlight the need to strengthen surveillance systems. The implementation of weather-based preparedness strategies is essential, including expanding preventive treatment coverage (SMC), deploying mobile health teams, and pre-positioning essential inputs.

**Supplementary Information:**

The online version contains supplementary material available at 10.1186/s12936-026-05833-z.

## Introduction

Climate change is increasingly influencing the dynamics of malaria transmission, particularly in the Sahel-Saharan regions where transmission is unstable and sensitive to climatic variations [1]. In northern Mali, increased extreme rainfall and flooding are promoting the proliferation of vectors and the emergence of malaria epidemics, often associated with a high number of severe cases and deaths. According to a report by the World Health Organization (WHO), climate change-related flooding may increase in malaria cases [2].

Climate change is increasing the frequency and intensity of extreme weather events worldwide [3]. According to the Intergovernmental Panel on Climate Change (IPCC), the Sahel-Saharan region is one of the region’s most vulnerable. The temperatures are expected to rise by at least 2 °C in the short term (2021–2040), at a rate 1.5 times higher than the global average [3], extreme rainfall events are also expected to rise, contributing to the occurrence of rain and river flooding [3].

In the Sahara, compared with other regions of Africa, forecasts of the frequency of climatic phenomena and the onset of epidemics are still poorly known. Mali suffered 15 floods between 1980 and 2024 [4]. The last year of flooding in the region, in 2024, caused extensive damage in all regions. According to the National Crisis Management and Coordination Center, the country recorded 729 reported flood events, leading to 47,306 building collapses, the destruction of 195,845 hectares of agricultural land, and the loss of 756,127 tons of cereals.

There were also 465,226 people affected, 33 health centers affected and 66,032 houses damaged [5]. This situation led to thousands of people being displaced, exposing them to health risks. In response to this situation, the government of Mali declared a state of national disaster (decree no. 2024–0485) [6].

Extreme rainfall during the first two weeks of August 2024 caused severe flooding in the Kidal region [7]. These rainfall and flooding expose the population to an increased risk of malaria in a context of prolonged insecurity and internally displaced persons, the situation has been exacerbated, especially as 83% of the population of Kidal has no access to a formal health center [8]. These circumstances not only complicated relief efforts but also confirmed the fragility of the region to the malaria outbreaks.

Despite the increase in extreme rainfall in the Sahel-Sahara region, studies describing the epidemic dynamics of malaria and the effectiveness of interventions during floods remain limited. This study aimed to describe the malaria outbreak associated with the 2024 floods in Kidal, Mali, and to assess the impact of extending Seasonal Malaria Chemoprevention (SMC) to broader age groups (0–15 years), as well as mobile health team interventions.

## Methods

### Study location

The study was carried out in the region of Kidal, located in the north Mali, comprising 4 health districts (HDs). The population of Kidal in 2024 was 106,775 (RGPH-2019) [9], spread over an area of 151,430 km^2^ and around 250 km from the Algerian border and 1,674 km from Bamako, the capital city of Mali. Located in the desert eco-climatic zone, the climate is characterized by wide variations in temperature: the average temperature is around 33 °C (with a minimum of 21 °C and a maximum of 45 °C). Average annual rainfall is 94 mm over 11 weeks [10], from June to September, and 135.11 mm over 15 weeks for 2024 [10], with 40.84 mm in 14 days, causing flooding in early August.

### Data collection and sources

#### Malaria cases and population

Cases of uncomplicated and severe malaria confirmed by Rapid Diagnostic Test (RDT) and by age group (0–11 months, 1–4 years, 5–14 years, > 14 years) from 23 May 2024 to 02 February 2025 were collected from the national epidemiological surveillance system, based on the District Health Information Software 2 (DHIS2) platform, which undergoes multi-level quality control. Data were aggregated at the district level and weekly.

Data describing number of confirmed malaria cases by RDT were extracted from the DHIS2 (National Information System) by the NMCP (National Malaria Control Program) on 7 February 2025. The population of the Kidal region was extracted from General Census of Population and Housing (RGPH), updated in 2019 [9]. These data was updated by multiplying the population from the 2019 by the annual population growth rate (3.3%) [9].

Historical additional data from 01 January 2021 to 22 May 2024 were also provided to facilitate the interpretation of the malaria study data.

#### Define the criteria for severe cases according to WHO 2022

Among the WHO severity criteria, we used on biological confirmation of *Plasmodium falciparum* infection (positive RDT and/or thick blood smear/thin film) combined with the clinical criteria as well as biological parameters [11]. The presence of at least one clinical and one biological sign (anemia or hypoglycemia), together with biological confirmation, was sufficient to classify the patient as a severe case.

#### Statement on the handling of missing data:

Indeed, during the epidemic period, reporting may have been interrupted, for example due to a loss of telephone coverage, but the data was subsequently recovered.

#### Intervention data

Intervention data from SMC and mobile health team were obtained from the National Malaria Control Program. A systematic monitoring system was in place during interventions.

#### Meteorological data

From 23 May 2024 to 02 February 2025, precipitation data (mm) were extracted from Tropical Rainfall Measuring Mission (TRMM) Rainfall Estimate L3 0.25 degree × 0.25 degree V7 (TRMM_3B42) produced at the NASA GES DISC [10] on a daily basis and then cumulated on a weekly basis.

Historical additional data from 01 January 2021 to 22 May 2024 were also provided to facilitate the interpretation of the precipitation study data.

### Data analysis

#### Descriptive analysis

The cases per 1,000 person-weeks was calculated for the whole period, for all confirmed malaria cases according to severity and age groups (0–11 months, 1–4 years, 5–14 years, > 14 years) [12]. The cumulative rainfall per week was calculated.

#### Spatial analysis

No personal data was included in the records. The spatial distribution of the population at risk of malaria was therefore estimated using WorldPop data. To this end, we applied the proportion of cases reported routinely at the heath district facilities to the estimated population living in the areas, extracted from WorldPop with a spatial resolution of 1 km^2^. This approach required us to assume a uniform spatial distribution of malaria risk across health districts and allowed us to estimate the theoretical spatial distribution of the population at risk of malaria.

#### Regression model

Rainfall was lagged by 5 weeks [13, 14] to account for vector development time and to optimize model fit by minimizing the Unbiased Risk Estimator (UBRE). Two binary variables were defined to represent the interventions. The two rounds of Seasonal Malaria Chemoprevention (SMC) were coded 1 for each week of round, including an effectiveness period of 28 days. The mobile health team interventions were coded 1 for each week of intervention. The time elapsed since its implementation was also included in the model. The full model, incorporating interventions, temporal trends, and rainfall, was used to simulate the time series of cases and assess its consistency with the observed data.

Interrupted time-series generalized additive models (GAMs) with a quasi-Poisson distribution were employed to estimate the effects of the interventions, adjusting for rainfall and using the logarithm of the population (log(pop)) as an offset. Analyses were conducted on data collected from May 2024 to February 2025.

Cases = offset(log(population)) + SMC + f(Time from SMC) + Mobile health team + f(Time from Mobile health team) + f(5 weeks lagged Rainfall) + random effect(Time), model = Quasi-Poisson.

#### Analysis software

Statistical analysis was carried out using R software (version 4.1.3). The map was produced using QGIS software (version 3.22.8).

### Ethical approval

Ethical approval for the present analysis was obtained from the Mali Ministry of Health (No.108/MSDSSG/PNLP, 05-Feb-2025) and Aix-Marseille University (Ref.2025-03-13-01). Only aggregated data were used, with no personal data nor identifiers involved.

## Result

### Historical data

**Fig. 1:**
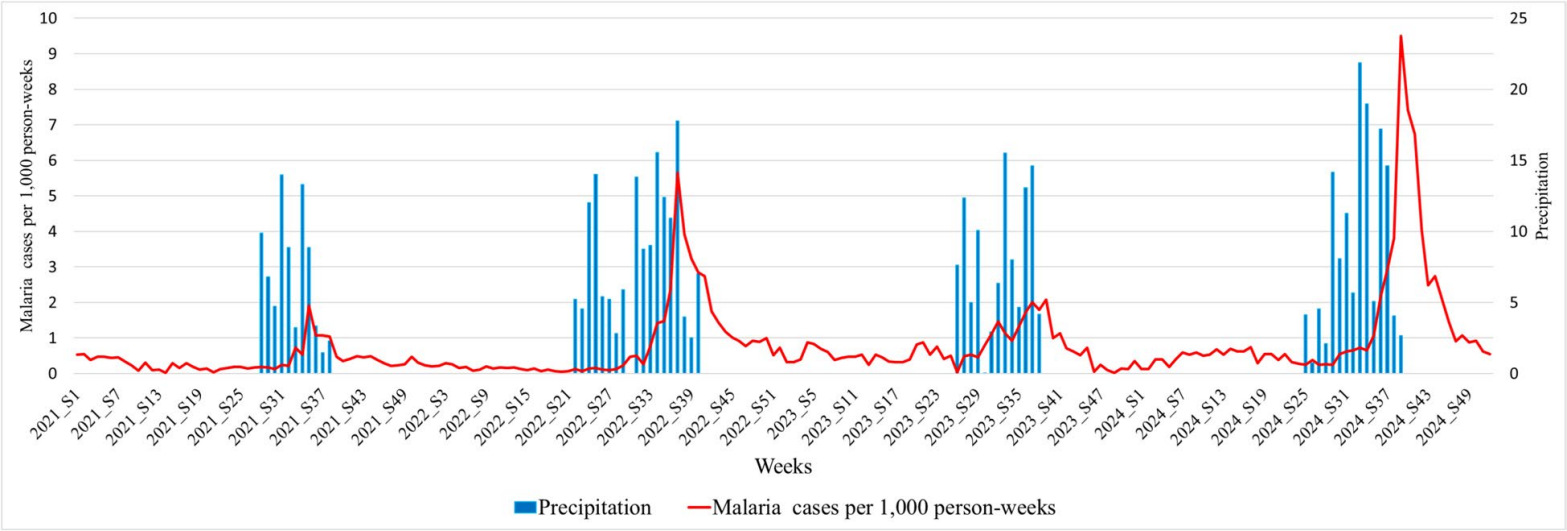
Temporal evolution of cases per 1000 person-weeks linked to rainfall from 2021 to 2024

Malaria cases per 1,000 person-weeks peaked during the rainy season, which began in week 21 and ended in week 40. In 2024, rainfall was more intense and concentrated, with a significantly higher peak in malaria cases per 1000 person-weeks than in previous years. This increase was faster, stronger, and longer (Fig.1).

The red curve represents the weekly malaria cases per 1000 person-weeks in the Kidal region from January 2021 to December 2024. The blue bars indicate cumulative weekly rainfall, expressed in millimeters (mm).

### General description of the malaria cases per 1000 person-weeks time series

**Fig. 2:**
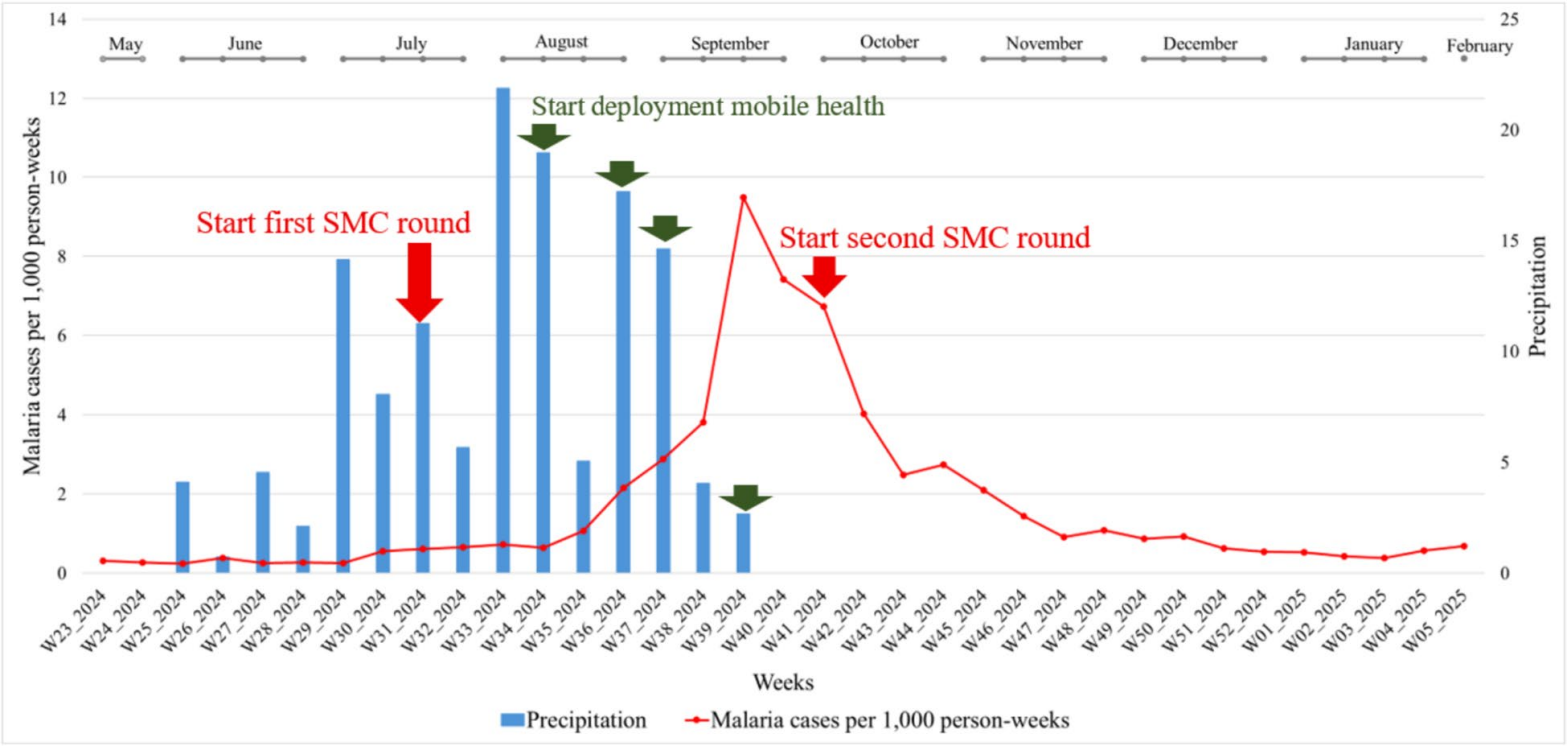
Evolution of weekly malaria cases per 1000 person-weeks and rainfall from S23 (May-2024) to S5 (February-2025)

From May 2024 to February 2025, 6311 malaria cases were confirmed, including 2310 severe cases. Rainfall began in week 25 and persisted for 15 weeks. The outbreak started in week 30, peaked in week 39 (1014 cases), and lasted 28 weeks with an overall cases per 1000 person-weeks of 1.688 (95% CI1.687–1.690). This incidence was markedly higher than in the previous three years, 2021, 2022, and 2023 (0.412, 1.154, and 0.706, respectively), according to a report from the National Health Information System. The second cycle of SMC was implemented just after the malaria of post-peak (Fig. 2).

The red curve represents malaria cases per 1,000 person-weeks in the Kidal region (6,311 cases reported by the national health information system) from week 23 in 2024 (May) to week 5 in 2025 (February). The epidemic peak was observed in September, in week 39, corresponding to the end of the rainy season. The blue bars represent cumulative weekly rainfall, expressed in millimeters (mm).

### Proportional contribution of each age group to total malaria cases:

**Fig. 3:**
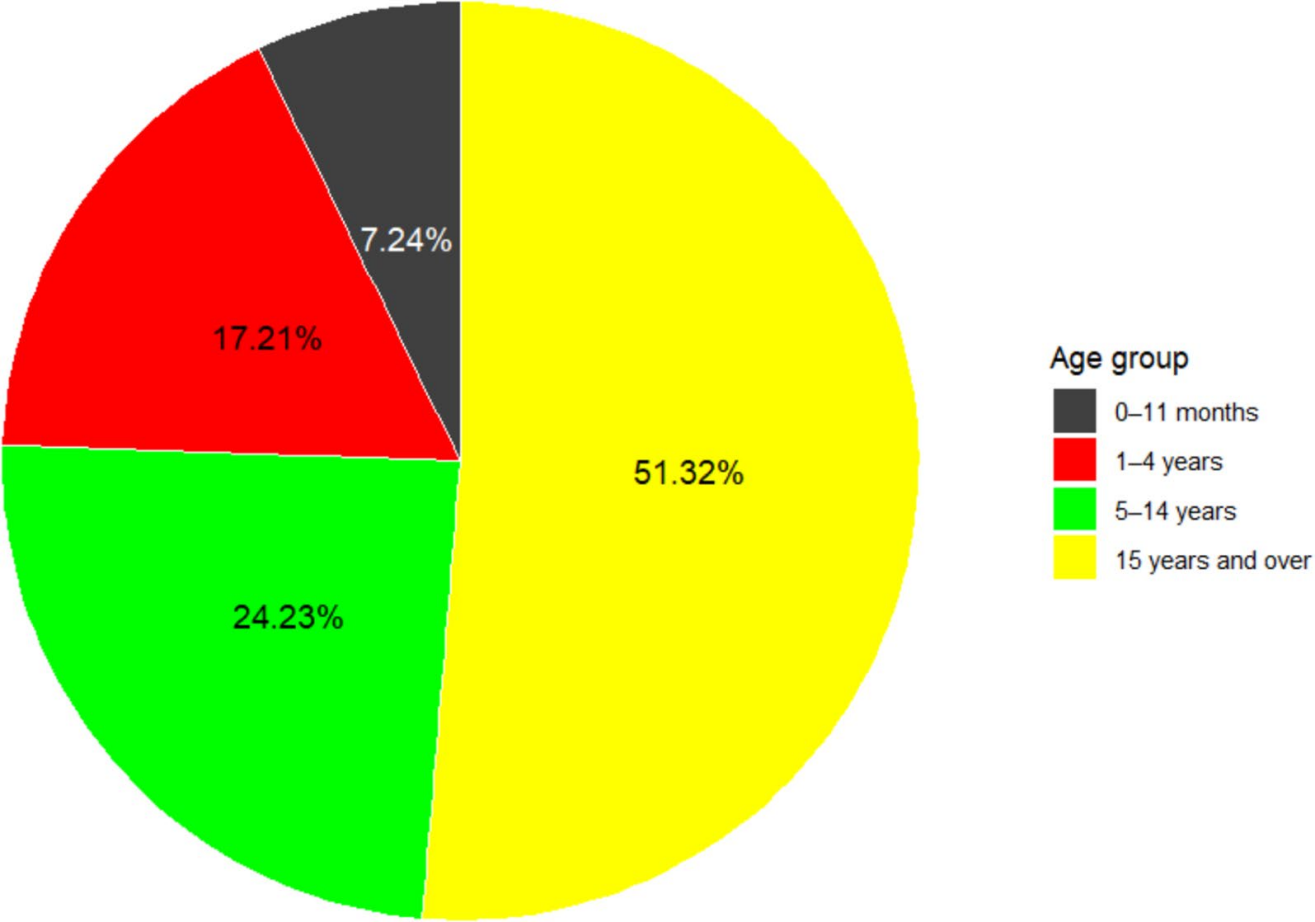
Proportional contribution of each age group to total cases

Figure 3 illustrates the proportional contribution of malaria cases from week 23 in 2024 (May) to week 5 in 2025 (February). The results indicate that individuals aged 15 years and older accounted for the largest share of reported cases (51.32%). Children aged 5–14 years represented nearly one quarter of cases (24.23%), followed by those aged 1–4 years (17.21%). Infants aged 0–11 months accounted for the smallest proportion (7.24%). Overall, the burden of malaria was higher among older age groups, suggesting greater exposure and/or increased vulnerability among adolescents and adults.

The Kidal region reported a total of 6311 malaria cases according to the national health information system. The yellow represents the proportional contribution of individuals aged 15 years and older, green corresponds to those aged 5–14 years, red to children aged 1- 4 years, and black to infants aged 0–11 months.

### General description of the time series of cases per 1000 person-weeks of uncomplicated malaria by age group

**Fig. 4:**
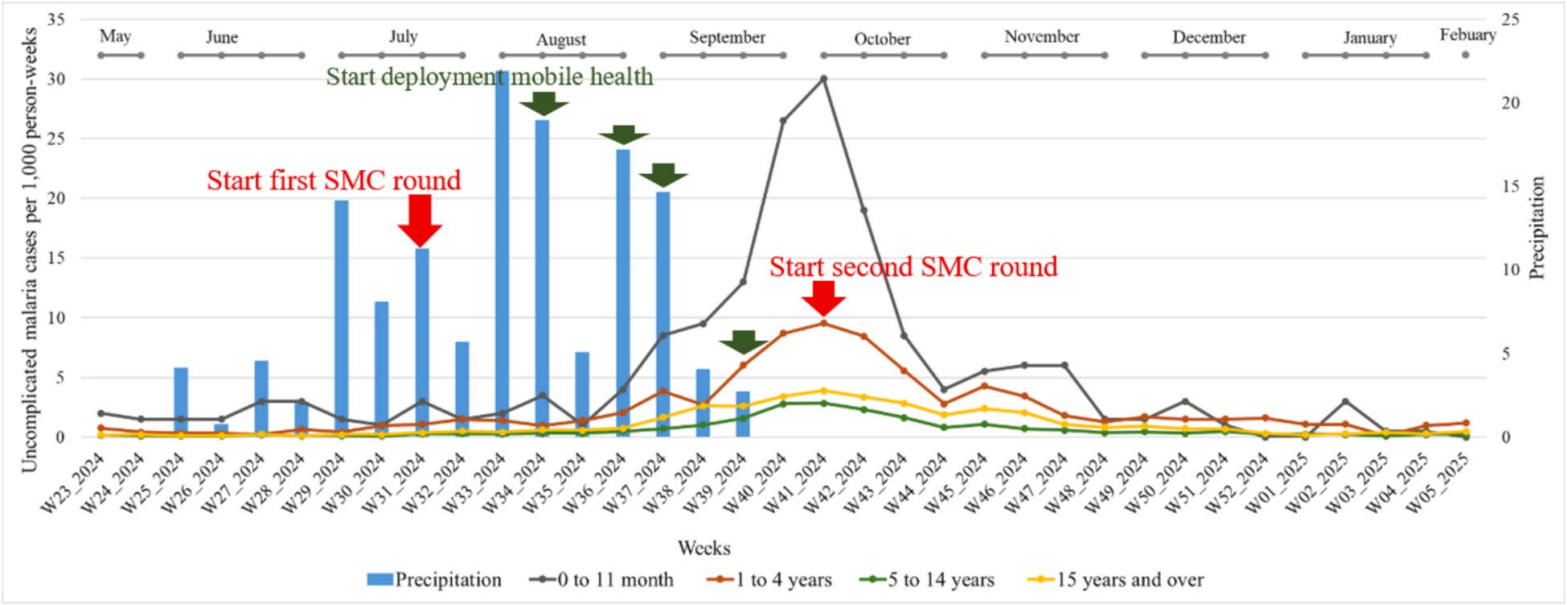
Trends in cases per 1000 person-weeks of uncomplicated malaria by age group and rainfall from S23 (May-2024) to S5 (February-2025)

From May 2024 to February 2025, 4,001 uncomplicated malaria cases were confirmed. The age groups most affected were children aged [0–11] months and [1–4] years, with cases per 1000 person-weeks of 5.04 and 2.35 respectively. On the other hand, the age group least affected was [5–14] years, with cases per 1000 person-weeks of 0.62 (Fig. 4).

Cases per 1,000 person-weeks in children aged [0 to 11] months are represented by the black line, in children aged [1 to 4] years are represented in red, in children aged [5 to 14] are represented in green, and in children aged 15 years and older are presented in yellow. Weekly cumulative precipitation (mm) is represented in the blue bar graph.

### General description of the time series of severe malaria cases per 1000 person-weeks by age group

**Fig. 5:**
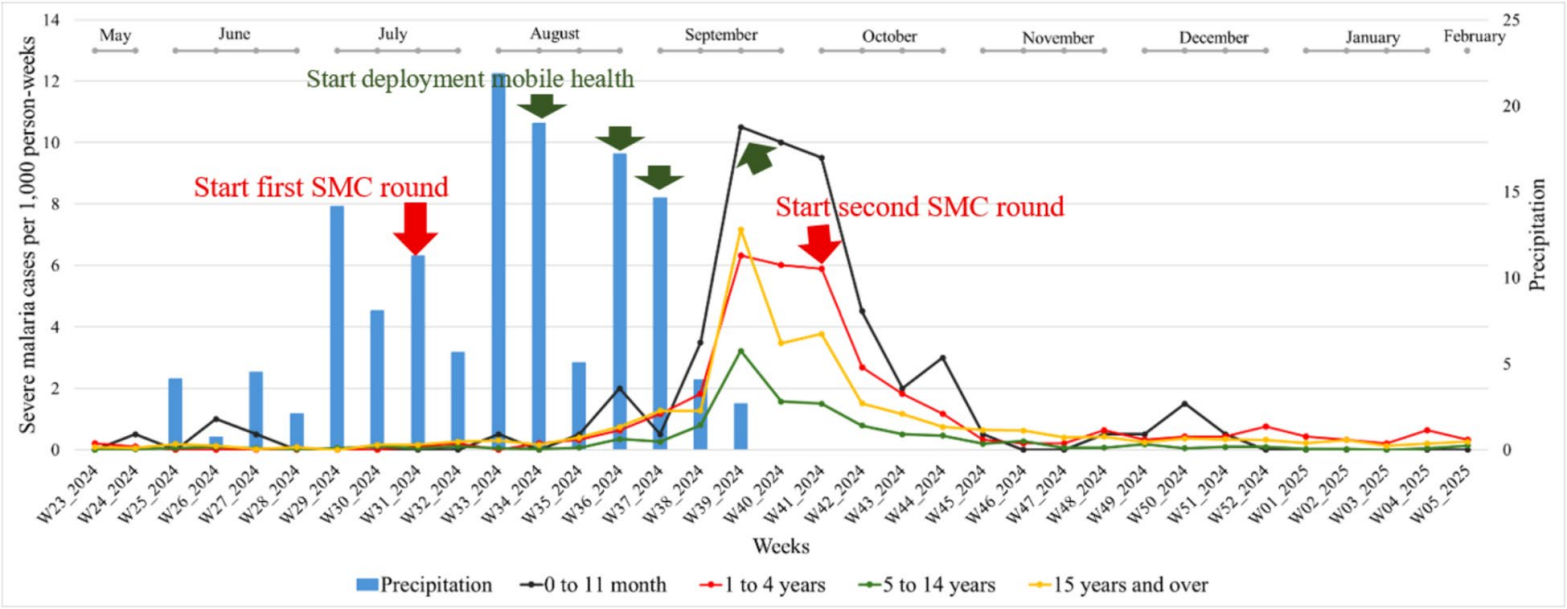
Trends per 1000 person-weeks of severe malaria by age group and rainfall from S23 (May-2024) to S5 (February-2025)

From May 2024 to February 2025, 2310 severe malaria cases were confirmed. The age groups most affected were children aged [0–11] months and [1–4] years, with cases per 1000 person-weeks of 1.48 and 0.97 respectively. On the other hand, the age groups least affected were those [5–14] years, with cases per 1000 person-weeks of 0.32. It is worth noting that people aged 15 and over presented many severe cases, with cases per 1000 person-weeks of 0.78, and a high peak of 355 severe cases, in week 39 (3rd week of September) (Fig. 5).

Cases per 1000 person-weeks are differentiated by age group: children aged [0–11] months are represented by the black curve, those aged [1–4] years by the red curve, those aged [5–14] years by the green curve and those aged 15 years and above by the dark yellow curve. Weekly cumulative precipitation (mm) is represented by the blue bar chart.

The age group of 15 years old and more years old had a proportion of severity rate of 0.593 with 1369 severe cases over a total of 2310 severe cases. higher than the other severity rates of 0.045 with 104/2,310 cases; 0.137 with 317/2,310 cases; 0.225 with 520/2,310 cases for [0–11] months; [1–4] years and [5–14] years, respectively.

### Map of the estimated population at risk per district

**Fig. 6:**
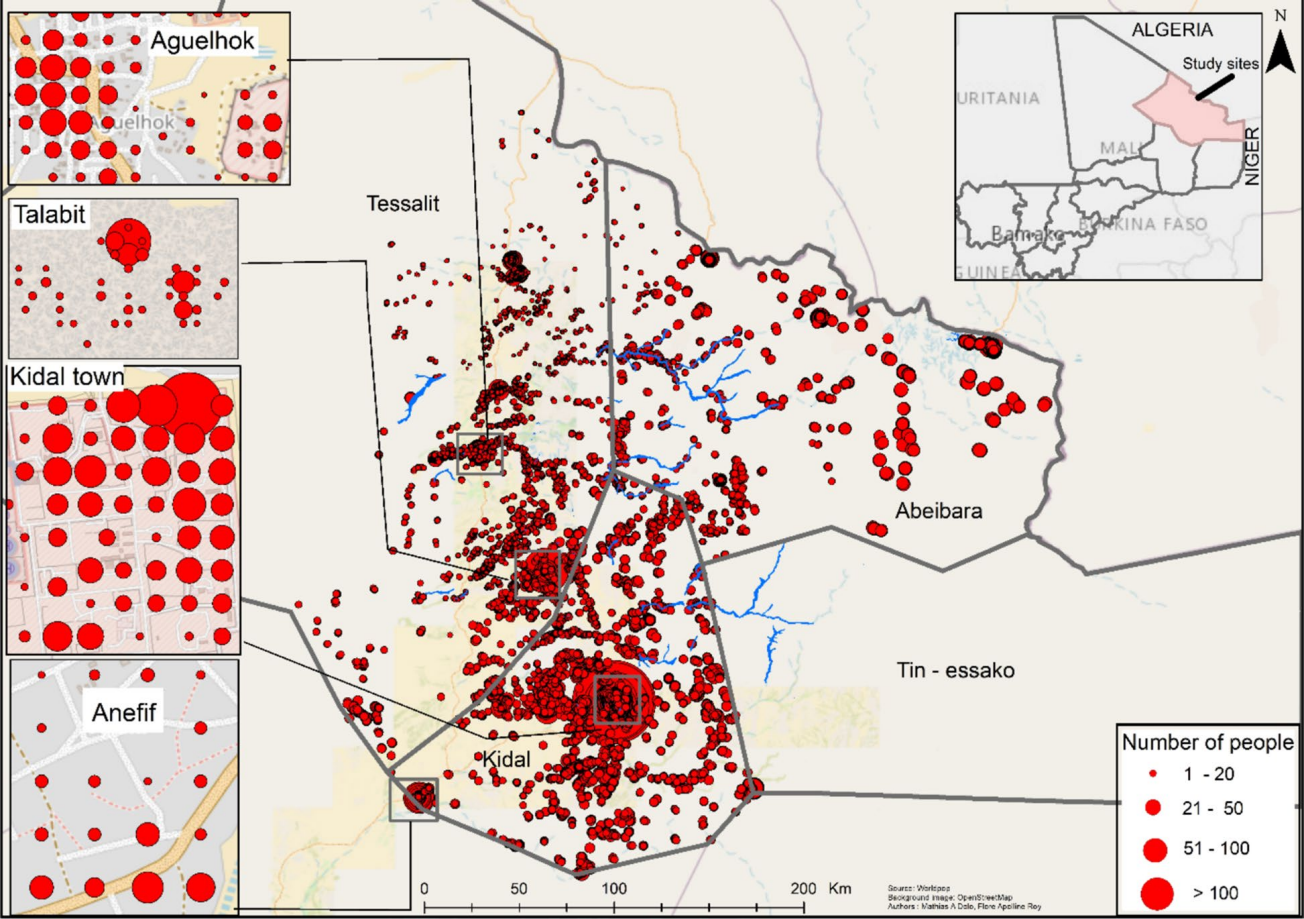
Map showing the distribution of estimates of the at-risk population in the different districts. The size of the red dots varies according to the estimated at-risk population, ranging from 1 to 154 people

Figure 6 shows the spatial distribution of the estimated population at risk per district. The highest level of population at risk was observed in the Kidal district, particularly in its central part, with a higher number of inhabitants. Conversely, the population at risk was low in the Tin-Essako district and in certain peripheral areas of Tessalit. High population at risk spots were also identified in the Abeibara district, particularly along the Algerian border.

### Response to the outbreak

Facing this malaria outbreak, the Kidal Regional Health Department, in collaboration with the National Malaria Control Program (Ministry of Health) and its partners, and the General Directorate of Health and Public Hygiene, undertook two types of interventions:

Mobilization of 36 community health workers was done for the 2nd seasonal malaria chemoprevention (SMC) campaign, aimed at children under 5 years old and extended to children up to 15. The first round, aimed at children under 5, took place before the epidemic, from July 29 to August 2, 2024 (31st week), treating 6236 children. The second round took place from October 6 to 10, 2024, corresponding to the 41 st week, and treated 27,334 children aged 0 month to 15 years.

Mobilization of seven mobile health teams, each comprising a driver, a doctor, a midwife, a nurse and a community mediator, were deployed to provide (free for all) diagnosis (RDT) and treatment (Artesunate inj 30 mg and 60 mg, Artemether inj 20 mg and 40 mg, quinine 400 mg and 200 mg and Artemisinin-based combination therapies (ACTs) Child, Adolescent and Adult, Sulfadoxine/Pyrimethamine, Paracetamol cp 500 mg and syrup) of malaria cases.

Uncomplicated cases were treated according to the national guidelines for the management of malaria using artemisinin-based combination therapies (ACTs) [15], whereas severe cases received injectable artesunate or other parenteral antimalarials, followed by oral treatment [15].

5 teams were deployed to remote areas in the Kidal district health units, and 2 teams were sent to Internally Displaced People (IPD) in the Kidal region. Over the whole period, 1561 cases of malaria were treated by mobile health teams.

The modeled outcome was the total number of malaria cases. The model showed a significant effect of both interventions on slowing the malaria epidemic, for extended SMC with an SIR of 0.5 CI95[0.33; 0.75] (*p* = 0.002), for the mobile health teams, an SIR of 0.48 CI95[0.28; 0.82] (*p* = 0.01). (supplementary file 1). The adjusted R^2^ of the model with intervention was 99.7%.

## Discussion

In the Kidal region, the floods of 2024 triggered a malaria epidemic of exceptional magnitude, associated with extreme rainfall, pre-existing malaria transmission, and a context of insecurity that weakened the health system. Children under five were the most affected, but unusual severity was observed in adults, highlighting their vulnerability in areas with an epidemic profile. Despite significant constraints, the interventions implemented, including extended seasonal chemoprevention and the deployment of mobile health teams, significantly reduced malaria-related morbidity.

The malaria outbreak that occurred in Kidal region between week 23 of 2024 and week 5 of 2025 was characterized by a very high incidence of 1.688 per 1000 person-weeks, markedly exceeding those reported between 2021 and 2023. The outbreak developed in the context of sustained heavy rainfall (135.11 mm over 15 weeks) [10], with a peak in August, creating favorable conditions for vector proliferation. Pre-existing low-level malaria transmission was likely amplified by these meteorological conditions. In addition, population concentration, particularly among nomadic groups and internally displaced people, increased exposure and vulnerability. The combination of environmental and demographic factors plausibly explains the intensity and persistence of the outbreak.

Several recent studies indicate that malaria remains prevalent among children older than 5 years, particularly in areas with high seasonal transmission [16, 17]. In Dangassa, extending seasonal malaria chemoprevention (SMC) to children aged 5–4 years proved to be feasible and well accepted [17], while in Kita and Bafoulabé, expansion to children aged 5–9 years led to a significant reduction in parasitemia [16], highlighting the role of older children in residual transmission. These findings support the adoption by the National Malaria Control Program (NMCP) of more flexible, context-specific strategies, also based on the experience of implementing this strategy to fight the 2015 epidemic [18, 19]. Accordingly, recent WHO recommendations [20] have prompted an expansion of targeted age groups, up to 15 years in certain settings, notably in Mali since 2024, thereby strengthening the potential for sustained reductions in malaria morbidity.

Children under five were most affected, but the elevated severity in inhabitants > 15 years was a concerning result, contrary to the profile of malaria in endemic areas [21]. This result probably highlights low background immunity in unstable transmission zones, as noted by Nkumama and al. and Baraka et al. The burden of malaria and its evolution over time remain poorly understood in adults and need to be studied in depth in areas with epidemic profiles [22, 23]. This suggests also that new strategy should be systematically implemented in case of extreme rainfall, such as SMC extension, mobile health teams and free treatment for all. New strategies could be tested such as mass-drug administration in case of flooding, or distribution of diagnostic self-test and treatment kits [24] for remote areas.

The number of malaria cases is likely underestimated due to service interruptions for security reasons, difficulties in accessing care, and uneven coverage of chemoprevention. The limited presence of community health workers and logistical disruptions related to weather and security crises have also restricted the scope of interventions. Despite these limitations, health services were able to maintain care for the population and data was subsequently recovered.

Logistics represents a key factor in the implementation of malaria interventions in isolated settings such as Kidal region. Limited access necessitated the use of air transport to deliver essential supplies, while local distribution was ensured through vehicles rented by the government and technical and financial partners. Service interruptions led to an increase in severe cases, highlighting the importance of extending seasonal malaria chemoprevention (SMC) to children up to 15 years of age, consistent with evidence of SMC effectiveness in areas of seasonal transmission [25, 26]. The secure storage of RDT and treatment supplies and the deployment of mobile health teams using locally rented vehicles to reach remote areas and perform rapid diagnostic and treatment were essential to maintain continuity of care, in line with recommendations regarding mobility and access to healthcare in rural settings [27]. Finally, interruptions of telecommunications networks due to the security situation were partially mitigated through the use of satellite connections, illustrating the importance of innovative approaches for surveillance in fragile contexts [28].

The Kidal health district had the highest population at risk from this epidemic and is the migration center for nomadic populations and the three other districts of the region. This concentration of populations could potentially aggravate the situation, making the management of the health crisis more complex. These results corroborate with Guetiya W et al. findings showing that modifiable risk factors such as overcrowding were associated with an increased incidence of malaria in Nigeria [29].

The mobilization of the regional health department [7], under the national coordination, the second round of seasonal malaria chemoprevention was organized (week 41) from October 6 to 10, 2024, and mobile health teams were deployed in areas far from health facilities and in camps for internally displaced persons to provide free care from August 10 to September 20.

The results of the study model confirmed that the interventions substantially reduced malaria-related morbidity. These findings were consistent with those of previous studies, notably those by Konaté et al., Ekezie et al., and Kwiringira et al., which demonstrated that the timely implementation of malaria control interventions such as seasonal malaria chemoprevention, early case management, and improved access to diagnosis and treatment (free for all) led to a significant reduction in malaria-related morbidity [29–31].

The fight against malaria is going through a period marked by a series of crises that threaten to undo decades of progress [32]. Sahelian countries severely affected by the disease are facing serious budget deficits and unprecedented security and weather crises [32]. According to the IPCC report, Sahelian countries are expected to experience extreme rainfall and flooding due to both rain and river flooding [3].

National malaria control programs must now proactively integrate future risks associated with climate variability and implement appropriate adaptation measures. Strengthening weather surveillance systems and establishing early warning mechanisms based on forecasts of heavy rainfall and flooding can improve the anticipation of high-risk periods and enable timely public health responses, as highlighted by the importance of climate-based malaria early warning systems in predicting outbreaks and guiding control interventions [33]. These mechanisms should be fully integrated into operational plans, particularly through the pre-positioning of essential diagnostic and treatment supplies before the onset of the rainy season, as well as through the reinforcement of supply chains capable of responding rapidly to climatic alerts. In this context, strengthening human resources remains a critical lever, especially through the engagement, training, and support of community health workers, who have demonstrated their effectiveness in delivering malaria care and guidance at the community level [34]. In parallel, the expansion and intensification of preventive interventions, such as seasonal malaria chemoprevention (SMC), are strongly warranted. Systematic reviews and implementation studies have shown that SMC significantly reduces the incidence of clinical malaria, and that its effectiveness can be optimized by adapting the number of cycles to the duration of the transmission season [35], extending SMC to broader age groups and implementing closely spaced campaigns could further maximize coverage and enhance impact [35]. In addition, in case of flooding, effective communication strategies, particularly through radio and television campaigns, are essential to raise public awareness and strengthen adherence to preventive measures. Finally, innovative and complementary approaches, such as self-administered test-and-treat kits or context-specific mass drug administration campaigns, could help limit the scale of epidemics and strengthen health system resilience facing malaria outbreaks associated with extreme weather events.

## Conclusion

In conclusion, the flooding that occurred in Kidal region was associated with a substantial increase in malaria incidence, disproportionately affecting children and adults from populations with low prior exposure to the disease. These findings, which may be extrapolated to other Sahelian regions facing similar environmental and security constraints, underscore the need to strengthen anticipatory and preventive approaches. The integration of weather data into malaria early warning systems, enhanced intersectoral collaboration between meteorological and health services, and improved health system resilience in fragile settings particularly through the deployment of community health workers are essential. Preventing and mitigating future epidemics will require comprehensive preparedness strategies, including the expansion of seasonal malaria chemoprevention, the deployment of mobile health teams, and the pre-positioning of essential diagnostic and treatment supplies. Finally, further research is needed to develop and validate weather-based early warning systems and to assess the effectiveness of innovative interventions in controlling malaria epidemics associated with extreme climatic events.

## Supplementary Information

Additional file 1.

## Data Availability

The database can be shared for use by other researchers with the authorization of the Malian Ministry of Health.
