# Supplementary material for: Extreme rainfall, flooding and malaria in the Sahara: outbreak analysis in Kidal, Mali 2024
Source: Malar J. 2026 Mar 6;25:158. doi: 10.1186/s12936-026-05833-z (PMC13078071; doi:10.1186/s12936-026-05833-z)
Supplement: Supplementary file 1 — Additional file 1. [file 12936_2026_5833_MOESM1_ESM.docx]

| **Interventions** | **SIRs (IC 95 %)** | **p-values** |
| --- | --- | --- |
| SMC | 0.50 (0.33 - 0.75) | 0.002 |
| Mobile health team | 0.48 (0.28 - 0.82) | 0.01 |
| Time since SMC |  | 0.03 |
| Time since mobile team |  | 0.44 |
| Rainfall |  | < 0.001 |

**Supplementary file 1:**

The modeled outcome was the total number of malaria cases. The model showed a significant effect of both interventions on slowing the malaria epidemic, for extended SMC with an SIR of 0.5 CI95[0.33; 0.75] (p = 0.002), for the mobile health teams, an SIR of 0.48 CI95[0.28; 0.82] (p = 0.01).
